# Using Mobile Health and the Impact on Health-Related Quality of Life: Perceptions of Older Adults with Cognitive Impairment

**DOI:** 10.3390/ijerph17082650

**Published:** 2020-04-13

**Authors:** Line Christiansen, Catharina Lindberg, Johan Sanmartin Berglund, Peter Anderberg, Lisa Skär

**Affiliations:** Department of Health, Blekinge Institute of Technology, SE-371 79 Karlskrona, Sweden; catharina.lindberg@bth.se (C.L.); johan.sanmartin.berglund@bth.se (J.S.B.); peter.anderberg@bth.se (P.A.); lisa.skar@bth.se (L.S.)

**Keywords:** aging, cognitive impairment, health-related quality of life, health technology use, phenomenography

## Abstract

Digital health technologies such as mobile health (mHealth) are considered to have the potential to support the needs of older adults with cognitive impairment. However, the evidence for improving health with the use of mHealth applications is of limited quality. Few studies have reported on the consequences of technology use concerning the older adults’ quality of life. The purpose of this study was to describe perceptions of mHealth and its impact on health-related quality of life (HRQoL) among older adults with cognitive impairment. The study was conducted using a qualitative design with a phenomenographic approach. A total of 18 older participants with cognitive impairment were interviewed. The interviews were analyzed in order to apply phenomenography in a home-care context. The results showed variations in the older adults’ perceptions that were comprised within three categories of description; Require technology literacy, Maintain social interaction, and Facilitate independent living. In conclusion, the development and design of mHealth technologies need to be tailored based on older adults´ needs in order to be understood and perceived as useful in a home-care context. For mHealth to support HRQoL, healthcare should be provided in a way that encourages various forms of communication and interaction.

## 1. Introduction

In an aging society where the requirements for extended care services are a prerequisite to meet the needs of older adults, the adoption of digital health technologies such as mobile health (mHealth) is seen as a solution [1,2]. Primarily for the optimization of support services but also to improve physical and mental wellbeing among older adults [3]. At the same time, there is still a lack of studies in the aging and technology field that include older adults with cognitive impairment [4].

Cognitive impairment is a condition that is often associated with dementia, which is a multifactorial disorder characterized by a progressive deterioration in memory and other cognitive domains [5]. There are different levels of difficulty related to the condition. Research [6] has shown that regardless of the level of cognitive impairment, both people with mild cognitive impairment and dementia experience cognitive changes that can be burdensome and change activity patterns over time, leading to consequences affecting their health and quality of life (QoL) [7]. Health is universally fundamental to individual wellbeing, personal achievements, and satisfaction. It is also embedded in notions of participation [8]. According to Baernholdt et al. [9], there is a strong association between health and QoL among older adults. Self-rated health has been shown to be strongly associated with subjective views of QoL in people with dementia while it has been concluded that QoL in people with dementia involves both cognitive function, activities of daily living, social interaction, and mental health [10,11]. This demonstrates that the relationship between health and QoL is complex and can be understood in different ways. Thus, the concept of health-related quality of life (HRQoL) seems more appropriate to use since it is limited to focusing on the effects of health and illness on QoL [12] and will therefore further be used in this study when examining the perceptions of mHealth. 

When examining mHealth, there are various definitions that can be used to describe its characterization. The Global Observatory for eHealth of the World Health Organization (WHO) [1] defines mHealth as “medical and public health practice supported by mobile devices, such as mobile phones, patient monitoring devices, personal digital assistants, and other wireless devices”. However, what distinguishes mHealth from other digital health technologies is its ability to provide mobile self-care, which enables people to monitor their health data without a clinician´s assistance and to communicate and interact remotely. Another definition of mHealth that has been described in relation to health communication is “the use of mobile and wireless technology devices for health-related interventions that seek to improve patient and public health outcomes” [13]. Compared to the other definition, the latter one which puts emphasis on technology as supportive at an individual level rather than organizational, are more suitable to use when evaluating subjective effects of mHealth interventions as similar to the aim of this study.

Previous research [3] has provided empirical evidence of the effectiveness of physical and mental health interventions using mobile applications. When examining mHealth technologies to support older adults with cognitive impairment, research has demonstrated that it can offer support in daily activities, relationships, memory, leisure activities, health and safety [14]. In addition, self-monitoring of health-related issues via mHealth technologies might increase the empowerment and engagement of older adults and thus support person-centered care [15]. However, it can be argued that technology development mainly has been focused on solutions to support safety needs in older adults with cognitive impairment [16,17] while less attention has been given to how the technology can be used to support individual needs and promote self-management in health. Based on the results from a systematic review [18] focusing on health outcomes and efficacy of mHealth applications in adults with cognitive impairment, the evidence for improving health with the use of mHealth applications is of limited quality. Few studies [19] have reported on the consequences of technology use concerning older adults´ QoL. Therefore, the aim of this study was to describe perceptions of mHealth and its impact on HRQoL among older adults with cognitive impairment.

## 2. Materials and Methods

### 2.1. Study Design

A qualitative design with a phenomenographic approach was used since the focus was to describe the variation in perceptions of mHealth and its impact on HRQoL among older adults with cognitive impairment, rather than identifying a common theme. Over the last decades, phenomenography has been used successfully both in medical and health care research [20] and is used to delineate the qualitatively different ways in which people experience a particular phenomenon [21]. Although the ontological and epistemological assumptions in phenomenography are linked to phenomenology, the approach differs in aims, goals, and methods [22]. Instead of aiming at understanding the essence of a phenomen as in phenomenology, phenomenography focuses on examining a collective human perception, which is essential in order to understand how a phenomenon is conceptualized by people [23]. Using a phenomenographic approach to study perceptions of mHealth and its impact on HRQoL may contribute to an increased understanding of its importance for future development and design of mHealth aiming at support HRQoL for older adults.

### 2.2. Context

The present study uses participants from an ongoing research project, The Support, Monitoring, and Reminder Technology for Mild Dementia (SMART4MD) [24]. The project is a multicenter randomized controlled trial aimed at investigating the effects of an mHealth intervention to improve the HRQoL of older adults with mild cognitive impairment and of their informal caregivers. All the older adults included in the project were community-dwelling and had a close relative or family member who served as their informal caregiver. In this sense, they had a close social network to be supported by, and therefore, none of the older adults were in social isolation even if some of them were living alone. The inclusion criteria were based on the mini-mental state examination (MMSE) where the older adults needed to score between 20–28 points to be included [24]. In the present study, an MMSE score of 20–26 points was used. The MMSE instrument contains questions regarding memory, learning, orientation etc. and scores from 0–30 points, where 26 points or less indicates cognitive difficulties [25]. Furthermore, to be included the older adult should be aged 55 or above, receive no formal care and experienced difficulties in recall for the last 6 months. Those who scored 11 or above on the Geriatric Depression Scale (GDS) or had a life expectancy of three years or less were excluded from the trial. All participants enrolled in the trial, has been given information about requests to participate in supplementary interviews as part of their informed consent.

### 2.3. Recruitment and Participants

The study sample, consisting of 18 older participants with a condition indicating mild cognitive impairment or mild dementia, was recruited from one of the SMART4MD´s clinical sites located in southern Sweden through telephone contact. The same inclusion and exclusion criteria, as used in the project described above, were used for this study with an exception for the MMSE score. A purposive sampling strategy [26] in regards to gender, age and MMSE was used to establish an extensive and varied reflection of the phenomenon. According to Larsson and Holmström [22] a large number of phenomenographic studies have shown that data collection from 20 participants is usually enough to discover all the different ways of understanding the phenomenon in question. In this study, no new perceptions were discovered after analyzing fourteen interviews. Hence, the study sample of 18 older participants were considered enough. The study sample comprised 12 men and 6 women, range 71–82 years (median = 78) (Table 1).

### 2.4. Data Collection

Individual semi-structured interviews were conducted, allowing the researcher to develop in-depth accounts of experiences and perceptions with individuals (Cousin, 2009). During interviewing, verbal probing with a concurrent approach was adopted, which helped to focus attention on pertinent issues without interfering with the actual process of responding [27]. All interviews were conducted for 1 month in the autumn of 2018. Pilot interviews with three volunteer participants were performed in advance to ensure the clarity of the questions during the interview and to train in probing techniques. Clarity of specific questions was adjusted accordingly. To ensure that the participants were focused on the phenomenon to be investigated, all interviews began with a short description of the meaning and purpose of mHealth (Appendix A), followed by a central open-ended question: “How do you perceive mHealth based on this description?”. Further, five more questions concerning their interactions of mHealth and personal experience of using mHealth were asked (Appendix B). Follow-up prompts such as “how” and “when” were used to elicit and clarify the participant’s answer. The majority of the interviews were carried out in the participant’s own home, while three interviews were conducted in the clinical research center based on the participant’s own choice. The time length of the interviews varied between 15 and 40 min (median = 28.5). All interviews were audio-taped and transcribed verbatim.

### 2.5. Data Analysis

The concept of perceptions, should according to Marton and Booth’s [23] definition, be understood as to how a phenomenon is experienced and that there are different ways in which a group of people understand a phenomenon. This description was used as a basis in the analysis of this study. The analysis was conducted jointly by the authors LC, CL and LS in consecutive steps in accordance with Sjöström and Dahlgren’s [28] revised guidelines based on the analysis process originally developed by Dahlgren and Fallsberg [29]. In the first step, the authors read each transcript individually to become familiar with the content. Identification of the most significant elements in relation to the purpose were then summarized and condensed from each transcript. In accordance with the following steps described by Sjöström and Dahlgren [28], the condensed responses were grouped into preliminary categories based on differences and similarities followed by a comparison aimed to establish borders between the categories. When the categories were clearly defined as different perceptions of understanding of the phenomenon, they were named to emphasize their essence. Based on a constant discussion and interplay between these steps, the authors created three descriptive categories. As a final step, a contrastive comparison was made describing the unique character of each category and the resemblances between them.

### 2.6. Ethics

The study was performed in compliance with the ethical guidelines of the Declaration of Helsinki [30]. The SMART4MD study has been approved by the Regional Ethical Review Board in Sweden (LU No. 650-00 and No. 744-00). Before entering the SMART4MD study, informed consent was obtained from each participant. Additional oral consent was obtained in conjunction with the interviews in this study. Further, to ensure confidentiality, each transcript was assigned a pseudonym in the form of a number (i.e., 1, 2, 3) that could be linked to the original transcript.

## 3. Results

The result of this phenomenographical study showed variations in the older adults’ perceptions of mHealth and its impact on HRQoL. The different variations are reflected in the following three categories of description; Require technology literacy, Maintain social interaction and Facilitate independent living (Table 2). The contrastive comparison between the categories showed that there were resemblances between two of the categories, where mHealth was perceived to be a supportive tool in everyday life that had an impact on the HRQoL of the older adults, but this was dependent on their technology literacy which was a prerequisite for the utility of mHealth. The categories are described below, accompanied by quotes that elucidate the responses of the older adults.

### 3.1. Categories of Description

#### 3.1.1. Require Technology Literacy

This descriptive category includes perceptions about usage, management, understanding, and access to mHealth. In terms of usage, the need for usability of mHealth was emphasized as essential and described as a demand. ”If I have pain in my hip, you cannot look in an application …it is not useful”. (13) Comparisons between telenursing and physical healthcare appointments were made, where the latter was preferable among most of the older adults. “ It is great that you can get quick help, but it requires that you can access it …that it is useful …you want to visit the doctor you had met before and got to know a little”. (3) Further, difficulties in using and managing mHealth were the most emphasized among older adults. Several of the adults considered themselves lacking both technical skills and sufficient experience of using mHealth. Besides, they felt that they could not entirely rely upon and feel safe when using mobile technology. Concerns about being afraid, regarding privacy and security, when searching for information on the Internet or sharing information on social media was described among the adults. They described this as both “dangerous” and “very risky” in case they got hacked while using banking services or misplaced the device somewhere. Difficulties in managing mobile technology were also perceived as time- and energy-consuming. Desires to participate in a technical course or having someone nearby, able to show them how to manage the device or ask questions whenever they needed help using the technology, was described among several adults.

Furthermore, mHealth was perceived as a technological solution that the older adults neither were interested in nor needed. Many of the adults described that they used mHealth mostly as a complement to become more engaged in daily life, but that they still felt they could manage to live without. They perceived that it was difficult to understand the need for mHealth and manifested a lack of perceived interest.

I do not understand what’s so interesting that you need to be connected night and day. There are newspapers and there are televisions that talk about the world in general …it’s enough if I need to call someone and ask for help, I am more than satisfied with that. (17)

It is probably good if you become familiar with it properly but I have not got that far yet that I need this thing with mHealth. (4)

Concerns about being dependent on technology that requires constant usage, in order to get the needs-adapted support, were also described along with more technical concerns such as losing the entered information. Therefore, many of the older adults preferred to use a regular paper calendar instead. Further, access to mHealth was described in relation to the cost of technology. The technology was perceived as a matter of cost, where the older adults described technology as “expensive” to purchase. Some of the older adults also described their concerns about not having enough money to purchase the technical equipment required to benefit from mHealth.

The unique character in this descriptive category was the contrasts in the older adults’ technology literacy. They described differences in their ability to use, manage, understand, and access mHealth which indicated that mHealth as technology literacy, therefore, had no impact on the older adults’ HRQoL.

#### 3.1.2. Maintain Social Interaction

This descriptive category includes perceptions about using mHealth for communicating with significant others to create feelings of safety and to stay informed on family matters and news. Many of the older adults perceived that mHealth facilitated communication with relatives and acquaintances, mainly by making oneself available all the time. They described that they mostly used their mobile phones to communicate by making phone calls or sending and receiving a text or multimedia message (SMS or MMS). Using their mobile phone to communicate was perceived as both “practical” and a “faster way of communicating” since they could bring it with them whenever they were going somewhere. Also, feelings of security were embedded in the use of mHealth. They described it as a technological solution that made them feel secure whenever they felt the need to call or contact someone if they needed help or if something were about to happen.

It doesn’t matter what I do, I always carry it with me, always actually. So, I am very easy to reach …I guess that’s the health I receive through my phone. (6)

I always bring it (the mobile phone) with me whenever I go somewhere…if anything would happen, I have it with me …it is an aid in this way. (15)

To a great extent, the older adults perceived mHealth as important for getting in touch with significant others, which was described in relation to communication through social media. Using social media, such as Facebook, was perceived as an alternative to keep in contact and stay informed about family matters and news in the outside world when physical contact was not possible. They described that they used either their smartphone or tablet for accessing social media and exchange information.

The unique character in this descriptive category was the perception of mHealth as a supportive tool that maintained social interactions, which provided a sense of security to the older adults’ HRQoL. Experiences about being able to keep in contact with people in everyday life, communicating with friends and their beloved ones as well as being able to participate in social events were described among the older adults.

#### 3.1.3. Facilitate Independent Living

This descriptive category includes perceptions about using mHealth for supporting recall in memory, health monitoring during illness, and feelings of wellbeing. During the interviews, the older adults described difficulties in recall, which sometimes seemed to create difficulties in their everyday life. mHealth was perceived to be supportive for recalls in several different ways, which they much appreciated as part of independent living. They used either their mobile phone or tablet for writing notes, setting reminders for appointments, taking photos with the camera function or receiving alerts from care units such as the dentist. In addition, they used game applications to play various cognitive games.

Further, mHealth was perceived as useful in supporting feelings of wellbeing and managing their health. Feeling physically and mentally well was considered essential for their independent living. Feelings of wellbeing were described in terms of health and illness, where the latter was perceived in relation to the absence of disease. The older adults described that maintaining health and having an absence of disease even though you are old was a prerequisite for experiencing a good QoL. Besides, the wellbeing of their significant others was mentioned as an essential aspect of their own QoL. In order to support health and wellbeing, they sometimes used mobile technology for searching health-related information on the Internet. In some cases, they also used mobile technology for health monitoring during illness, where the use of a health application for self-monitoring of diabetes was brought up as an example. The health application was perceived as support in keeping track of the glucose level by facilitating the daily registration.

I have had diabetes for 10 years and I started to use some mediocre apps early in the days … it’s a way of keeping track of my blood sugar level and because of that, I have never gone badly …I feel safer because I know how my glucose level varies over time. (18)

The unique character in this descriptive category was the perception of mHealth as a way to control everyday life and thereby support an independent living, which was interpreted as being related to the HRQoL of the older adults.

## 4. Discussion

The different ways the older adults in this study perceived mHealth were mainly described as requiring a technology literacy, which can be seen as a prerequisite for the utility of mHealth among this study sample. In its broadest sense, technology literacy can be defined as an individual’s ability to use, manage, understand and access technology [31]. This is related to digital literacy, in that when an individual is proficient in using computers and other digital devices, digital literacy gives them the ability to use the Internet and use information via various digital platforms such as a web browser, newspapers and social media sites. Using mobile technology to seek knowledge about health information on the Internet and apply the knowledge gained to address or solve a health problem, also referred to as electronic health literacy (eHealth literacy) [32], can be a resource to facilitate independent living. However, as demonstrated in the results of this study, the ability to use, manage and access mHealth among older adults with cognitive impairment varies significantly. Several of the older adults considered themselves lacking both technical skills and sufficient experience of using mHealth. Also, concerns about being afraid, concerning privacy and security, when using mHealth were emphasized. The balance between the benefits of using technological solutions and the fundamental rights of older adults has shown to be of importance among community-dwelling older adults. Studies [33] have also shown that older adults with cognitive impairment seem to prefer standard and less complicated health technology due to different levels of complexity affected by several habit aspects. According to Lupton [8], who critically addresses a range of compelling issues about the rise of digital health technologies, less knowledge, and fewer skills in using digital health technologies can exacerbate existing social inequities that contribute to weaker health status and higher levels of poor health. As more and more of society’s welfare services within Scandinavia are being encircled by digital information channels, the requirements for a digital literacy increases in order to be able to actively participate in the society [34]. Since the ability to use, manage and access mHealth in older adults with cognitive impairment was found to be varied, they are at higher risk of digital exclusion. However, when looking at patterns of digital health technology use, this issue might not solely be linked to the population of older adults with cognitive impairment. Research [35] comprised of younger adults with low socioeconomic status and who lacked social contacts who could teach them how to use digital health technologies, expressed a lack of confidence in skills and literacy level to access and understand health and medical information online. Other studies [36] have identified physical ability, motivation, cognition and perception as aging barriers influencing the usability of mHealth. Against this background, it can be argued that the use of digital health technologies can be seen as a dimension of social inequality. Nevertheless, the results of this study imply that the technology literacy related to the use of mHealth among older adults with a cognitive impairment needs to be addressed at a societal level to reduce social inequalities and avoid the risk of digital exclusion.

Furthermore, in the category of technology literacy, some of the different perceptions might be a result of an underlying complexity pertaining to the notion of the concept of HRQoL. Within the scientific literature, the concept of QoL is often used to describe wealth, happiness, meaningfulness, and freedom of action, which can all be seen as the hallmarks of a good life. But in relation to health, the concept is used to measure a variety of different aspects such as health status, physical functioning, psychosocial adjustment, symptoms, wellbeing and life satisfaction [37]. While measurements of QoL are relevant to the evaluation of outcomes of health and social care interventions and in relation to conditions that can affect a person’s whole life, lack of precision in terminology about QoL has resulted in the use of the same terms to mean different things [12]. This may partly explain why many of the older adults in this study perceived that it was difficult to understand the need for mHealth in relation to their HRQoL and thus manifested a lack of perceived interest. From a broader perspective, this reflects issues related to the development and design of gerontechnology to meet the needs of an aging population. It has been discussed whether the need for the use of gerontechnology has emerged from older adults’ own needs or the needs of stakeholders (i.e., relatives, healthcare professionals) [38]. Within an aging population, older adults with cognitive impairment constitute a heterogeneous group where support needs to be based on the level of cognitive functioning. Previous studies [39] have described that most older adults with cognitive impairment reported that they need assistance in instrumental activities of daily living (IADL) and that higher rating on perceived QoL is correlated with higher MMSE value. Also, living at home and having the ability to perform activities of daily living independently has been shown to be associated with high HRQoL among this study population [40]. Although, the median value of MMSE for the participants in this study was rather high (MMSE 25), suggesting that they are still capable of coping on their own [6], their ability to independently perform activities of daily living are likely to decrease as the condition progresses and thereby increases the need for support and care. Therefore, the development and design of future mHealth technologies need to be tailored based on older adults’ needs in order to be understood and perceived as useful in a home-care context.

Despite the above, the results of this study also showed some perceptions of mHealth that had an impact on the HRQoL. The unique character that the use of mHealth was supportive of maintaining social interactions seemed to be the principal way of perceiving mHealth. Among these perceptions, interactions through social media were emphasized as a useful alternative to keep in touch and stay informed on family matters when physical contact was not possible. This illustrates that increased possibilities to communicate and interact may reduce feelings of loneliness and enhance HRQoL. Previous research [40] aiming at identifying factors associated with HRQoL in older adults with cognitive impairment has demonstrated that feelings of loneliness are associated with low mental health, which affects one of the two dimensions of HRQoL. However, there are studies [41] emphasizing that the use of social media is rarely considered as social interaction. Research [42] also describes that older adults in general, highly value human contact and that contact with another human being cannot entirely be replaced by technology. This does partly reflect what was described among the adults in this study, where they made comparisons between telenursing and physical healthcare appointments and preferred the latter. Thus, to support both physical and mental health, that is, the two dimensions of HRQoL, in older adults with cognitive impairment, it is vital that healthcare can be provided in a way that encourages various forms of communication and interaction. In this way, healthcare will be based on a person-centered approach that can promote self-management in health.

### Methodological Considerations

There are certain aspects of trustworthiness in this study that will be discussed in accordance with the concepts of trustworthiness outlined by Lincoln and Guba [43]. The establishment of the semi-structured interview guide used in this study can be seen as a demonstration of *dependability*. This became evident when some of the older adults had difficulties describing the relation between mHealth and their QoL. The use of the interview guide and the description of mHealth helped to maintain focus on the phenomenon while ensuring that all interviewees were given open-ended probes within the same theme. Further, in an attempt to increase the *credibility* of this study’s results, a varied sample selection that contributed to different ways of understanding the phenomenon investigated was deliberately recruited. Moreover, all interviews were conducted and transcribed by the first author, leading to an increased understanding of the material that was considered beneficial during the analysis process. To avoid influence from the researcher’s preunderstandings, all the consecutive steps in the analysis were conducted and discussed repeatedly between co-authors. Nevertheless, a possible limitation in this study can be referred to as the amount of gathered data material. Some of the interviews were quite short, between 15–20 min, which resulted in less extensive data. Even though a more extensive amount of data would have yielded more varied information that could lead to an in-depth understanding of the phenomenon, the short interviews were included considering they both strengthened the other descriptions and responded to the purpose of this study.

Furthermore, there seemed to be a relationship between the use of mHealth and the degree of cognitive impairment, indicating increased difficulty with usage. This relationship was illustrated during the interviews, where two of the older participants described that they were currently not using any mobile technology all, as their cognitive function has deteriorated rapidly. No efforts to further investigate this relationship was made. It can, therefore, be argued that the inclusion of these two participants’ perception constitutes a limitation for the *confirmability* of this study’s results, questioning whether their perceptions of mHealth were based on preconceptions or actual experiences of using mHealth. However, the motive for still including their perceptions was based on the fact that both of them had prior experience of frequently using mHealth. Besides, they contributed with valuable information concerning HRQoL for this population.

The results generated from this study were based on patterns recognized empirically both in this study and in related research [44] that focused on technology to support aging in place. According to Larsson [45], *transferability* of qualitative research results can be seen from several perspectives, including a generalization through recognition of patterns. Therefore, it is reasonable to assume that these results might be transferred to other older populations with chronic conditions (i.e., chronic heart failure, diabetes) in similar needs of supporting health and independent living. Although the results from this study cannot be applied directly into clinical practice [28], these results can be used as a foundation for future research focusing on evaluating the effects of mHealth among older adults with cognitive impairment.

## 5. Conclusions

This study showed that the use of mHealth is perceived as a multifaceted phenomenon that requires technology literacy to support independent living and social interactions in older adults with cognitive impairment. The results imply that the technology literacy level related to the use of mHealth among older adults with a cognitive impairment needs to be addressed on a societal level to reduce social inequalities and avoid the risk of digital exclusion. Further, the development and design of future mHealth technologies need to be tailored based on older adults’ needs in order to be understood and perceived as useful in a home care context. For mHealth to support HRQoL in older adults with cognitive impairment, healthcare should be provided in a way that encourages various forms of communication and interaction. Thus, the use of mHealth can to some extent support aging in place for this population.

Modern life requires dealing with technology to participate actively in society. As the technology literacy of mHealth in older adults with cognitive impairment varies, these perceptions highlight the importance of developing and designing mHealth technologies that are perceived as easy to use and useful in relation to the older adults’ needs. We, therefore, recommend future research to consider the needs related to different levels of cognitive impairment when examining the effects of mHealth technologies. Knowledge generated from such studies may have a greater influence on the adoption and use of mHealth among older adults with cognitive impairment.

## Figures and Tables

**Table 1:** Study population characteristics (*n* = 18).

Variable	Male *n*	Female *n*	Total *n*
Gender	12	6	18
Age groups			
70–75	4	2	6
76–80	6	-	6
81+	2	4	6
MMSE level *			
20	-	-	-
21	2	-	2
22	1	-	1
23	2	-	2
24	-	1	1
25	2	1	3
26	5	4	9

* MMSE Level [25]: Mini-mental state examination where 20–26 points indicate mild cognitive impairment.

**Table 2:** Overview of the categories of description with the number of responses per perception.

Categories of Description	Perceptions of Mobile Health	Number of Resonses
Require technology literacy	Need for usability Lacking technical skills Fearing use Desiring education or support Lacking need A matter of cost	8 27 17 15 29 7
Maintain social interaction	Facilitating communication Feelings of security Staying informed	38 12 6
Facilitate independent living	Supporting recall in memory Feelings of wellbeing Health monitoring during illness	7 5 2

## Appendix A

### Description of mHealth

Mobile health (mHealth) includes technology that is used to support people’s health and healthcare practitioners. Examples of such technology are mobile and wireless technology devices such as; mobile phones, computer tablets and personal (portable) digital assistants. Currently, mHealth is used to collect health data and track people’s health, but also to support patients in everyday life. An example of this is a person with diabetes who daily registers their blood sugar value to gain better control of their own health. It can also be about using mobile health applications * to communicate with their relatives. In previous research, mHealth has proven effective for increasing people’s opportunities for self-care. In addition, mHealth has the potential to cut waiting lists for healthcare appointments by reducing the need of physical healthcare meetings with professionals’, which in turn can contribute to reduced healthcare costs.

* Health applications are a collective name for a variety of applications “apps” that can be downloaded on mobile phones or tablets so that the user can more easily get an overview and be able to affect their own health and wellbeing.

## Appendix B

### Semi-Structured Interview Guide

1. How do you perceive mobile health based on this description?
2. Have you previously had contact with mobile health?
3. In which situations have you used mobile health?
4. In which interactions do you use mobile health?
5. What do you perceive as quality of life for you?
6. In what way does mobile health contribute to an increased quality of life for you?
